# Adverse Cardiac Events of Hypercholesterolemia Are Enhanced by Sitagliptin Administration in Sprague Dawley Rats

**DOI:** 10.21203/rs.3.rs-4075353/v1

**Published:** 2024-03-19

**Authors:** Henry A. Palfrey, Avinash Kumar, Rashmi Pathak, Kirsten P. Stone, Thomas W. Gettys, Subramanyam N. Murthy

**Affiliations:** Southern University and Agricultural and Mechanical College; Southern University and Agricultural and Mechanical College; Southern University and Agricultural and Mechanical College; Pennington Biomedical Research Center; Pennington Biomedical Research Center; Southern University and Agricultural and Mechanical College

**Keywords:** Cholesterol, Methionine, Cardiovascular, Sitagliptin

## Abstract

**Background:**

Cardiovascular disease (CVD) affects millions worldwide and is the leading cause of death among non-communicable diseases. Western diets typically comprise of meat and dairy products, both of which are rich in cholesterol (Cho) and methionine (Met), two well-known compounds with atherogenic capabilities. Despite their individual effects, literature on a dietary combination of the two in the context of CVD are limited. An additional interest was to investigate the cardioprotective potential of sitagliptin, an anti-type 2 diabetic drug. Thus, *we hypothesized that atherogenic feeding would result in adverse cardiac effects and would attenuate upon sitagliptin administration*.

**Methods:**

Six-week-old adult male Sprague-Dawley rats were fed either a control (Con), high Met (1.5%), high Cho (2.0%), or high Met (1.5%) + high Cho (2.0%) diet for 35 days. They were orally gavaged with vehicle (water) or *sitagliptin* (*100 mg/kg/d*) from day 10 through 35. On day 36, rats were euthanized, and tissues were collected for analysis.

**Results:**

Histopathological evaluation revealed a reduction in myocardial striations and increased collagen deposition in hypercholesterolemia (HChol), responses that became exacerbated upon sitagliptin administration. Cardiac pro-inflammatory and pro-fibrotic responses were adversely impacted in similar fashion. The addition of Met to Cho (MC) attenuated all adverse structural and biochemical responses, with or without sitagliptin.

**Conclusion:**

Adverse cardiac outcomes in HChol were enhanced with sitagliptin administration and such effects were alleviated by Met. Our findings could be significant for understanding the risk-benefit of sitagliptin in type 2 diabetics who are known to consume atherogenic diets.

## Introduction

Inflammation is considered as a cornerstone in many disease processes, particularly those of the cardiovascular system [1, 2, 3, 4]. Although protective, unfavorable outcomes could occur if inflammation persists for a long period of time, as seen in the case of atherosclerosis [5]. Atherosclerosis is a type of arteriosclerosis (hardening of arterial walls) that is characterized by fibrofatty lesion formation in arterial walls. This causes arteries to become stenotic, impeding normal blood flow, and resulting in a multitude of downstream adverse effects [5]. From the onset of the atherosclerotic process to advanced stages, where complete plaque formation is present in arterial walls (hallmark of CVD), the expression of proinflammatory (e.g., tumor necrosis factor alpha, *Tnfα*; interlukin-1 beta, *Il1β*; etc.) and other biochemical indicators are commonly observed [6]. Essentially, it is biochemical processes like inflammation or oxidative stress that precede adverse structural changes, as seen in fibrosis [7].

Western diets are believed to contribute to CVD as they largely consist of compounds like sugars, Cho, sodium, and saturated fats among others [8, 9, 10, 11]. Additionally, Cho and Met are compounds with atherogenic potential that are found in large quantities in meat, poultry, and dairy products [12, 13]. Where approximately 70% of Cho is synthesized *de novo*, the dietary Cho contributes to about 30% of total body Cho [14, 15, 16]. It is, however, the overconsumption of Cho in the human diet that has long been debated as a causative factor for CVD [17, 18, 19]. Initial reports of Cho as a contributing factor in CVD stem from results of the Framingham heart study of the 1960s [20]. Furthermore, elevated dietary Cho has also been associated with pro-inflammatory signaling in adipocytes, a situation that can adversely affect the heart in obese and diabetic patients [21,22].

Methionine is an essential amino acid that initiates eukaryotic protein synthesis and serves as a methyl group donor in DNA, protein, and other methylations [23, 24]. Defects in Met metabolism, deficiencies of vitamins B6, B12 or folate, or increased consumption could result in the accumulation of an intermediate compound, homocysteine (Hcy), in circulation and result in hyperhomocysteinemia; a noted risk factor for CVD [25]. Kilmer McCully, a pioneer of the Hcy theory, demonstrated that Hcy damages tissues by stimulating the release of cytokines, cyclins, and other mediators of inflammation and cell division [26]. Troen and coworkers have also reported an atherogenic effect of excess methionine [27]. Conversely, dietary Met restriction has been demonstrated to produce beneficial effects like improving insulin sensitivity and lipid profile and enhancing metabolic flexibility [28, 29, 30].

Strong evidence has surfaced in recent years that highlights an association between non-alcoholic fatty liver disease (NAFLD) and increased CVD risk [31]. In fact, atherosclerotic CVD has been considered to be the main cause for mortality in patients diagnosed with NAFLD [31]. The underlying mechanisms for the relationship between NAFLD and CVD are believed to be incompletely understood; however, inflammation, endothelial dysfunction, and dyslipidemia are positioned as significant factors [32]. Our lab previously observed hepatic inflammatory and oxidative stress responses in HChol, an observation that was exacerbated by sitagliptin administration [33, 34]. Sitagliptin (Januvia) is a type 2 diabetic drug currently in clinical use for the management of hyperglycemia, via dipeptidyl peptidase-4 (DPP-4) inhibition [35]. Independent of its hypoglycemic effect, sitagliptin has been shown to provide multiple health benefits such as attenuating heart and kidney failure and helping to improve neurological function [36, 37, 38]. Provided with our previous data, we advanced our studies and thought to investigate how the heart is affected upon atherogenic feeding and evaluate the cardioprotective potential of sitagliptin. *Therefore*, *we hypothesized that atherogenic diets would enhance cardiac inflammatory responses and administration of sitagliptin would alleviate such effects*.

## Materials and Methods

### Animals and Diets.

All animal experiments were performed according to the National Institutes of Health Guide for Care and Use of Experimental Animals. The protocol was approved by the Institutional Animal Care and Use Committee of the LSU Pennington Biomedical Research Center in Baton Rouge, LA. Six-week-old adult male Sprague-Dawley rats weighing 250–270g were obtained from Envigo RMS, Inc. (Indianapolis, IN). Purina #5001 Chow containing 25.05% carbohydrate, 24.1% protein and 11.4% fat and supplemented with 0.5% cholic acid and 2.0% maltose dextrin was used for the control (Con) diets. Dyets, Inc. (Bethlehem, PA) prepared the experimental diets by enrichment of the Con diet with 1.5 Met %, 2.0% Cho, and 1.5 Met % +2.0% Cho (MC). Rats were housed individually in cages with standard bedding in a temperature and humidity-controlled room with a 12-hr day/night cycle for acclimatization for one week. Food and water were provided *ad libitum*.

### Experiments

In the *first animal grouping*, rats were weight-matched and divided into four dietary groups (*Con*, *Met*, *Cho*, *MC*, n = 7 per group) and fed for 35 days. In the *second animal grouping*, rats were weight-matched and assigned to Con (n = 14), *Met* (n = 7), Cho (n = 7) and *MC* (n = 7) groups. On day 10, half the Con and all rats in Met, Cho, and MC were orally gavaged with an aqueous suspension of *sitagliptin* (*100 mg/kg/day*). The remaining Con rodents were orally gavaged with vehicle (water) to validate an expected null effect of drug with a normal diet. The diet and drug regimen continued for 25 days. In a *third animal grouping*, rats were weight-matched then assigned to *Con*, *Cho*, and *MC* groups (n = 16 per group); single Met group was omitted. This experiment was conducted to authenticate findings of the 1st and 2nd animal groupings, where diet and drug effects were assessed independently. On day 10, half the rats in each group were orally gavaged with vehicle, while the remaining half were administered *sitagliptin* (*100 mg/kg/day*) by oral gavage. The diet and drug regimen continued for 25 days. After a 4-hour fast on day 36, animals in each grouping were euthanized by CO_2_ inhalation (for respiratory arrest) followed by the collection of blood and heart tissues for biochemical analysis and histopathological evaluation.

### Measurement of body composition

Time domain-nuclear magnetic resonance (NMR) spectroscopy (Bruker Minispec, Billerica, MA) was used to measure body composition at weekly intervals. Calibration of the NMR instrument was achieved using appropriate fat, lean mass, and water standards per the manufacturer’s protocol. Body weight was taken weekly as well.

### Sample Collection

Initial (fasting) blood samples were collected prior to the start of each experiment via retro-orbital puncture under 2.0% isoflurane anesthesia, and final samples were taken by cardiac puncture following euthanasia with inhalation of CO_2_. Serum was separated from whole blood for metabolomic analysis, which was performed by liquid chromatography-mass spectrometry. Weekly blood samples were taken, via tail vein, for the measurement of blood glucose using a glucometer. A segment of the heart tissue encompassing the left and right ventricles was processed for histopathological evaluation and the remaining tissue was snap frozen in liquid nitrogen, then stored at −80 °C for subsequent biochemical analysis.

### Histopathology

Masson’s trichrome staining was performed to assess collagen accumulation occurring in heart tissue. Heart samples were fixed in 10% neutral buffer formalin and processed on a TissueTek VIP 6 Vacuum Infiltration Processor. Following fixation, they were embedded in paraffin and 5 μm sections were obtained for evaluation. Trichrome staining involved initially deparaffinizing and rehydrating slides through a series of descending alcohol washes (100%, 95%, 70%). They were then washed in distilled water and re-fixed by incubation in Bouin’s solution (75mL Picric acid-saturated; 25 mL formaldehyde-37%; 5mL glacial acetic acid) for 1 hour at 56°C. The purpose of re-fixation was to improve staining quality. Slides were stained by placement in a working solution made from two Weigert’s Iron Hematoxylin stock solutions. Next, they were placed under running warm tap water for 10 minutes for rinsing, washed in distilled water, and stained once more by placement in a Biebrich scarlet acid fuchsin solution for 10 minutes. For the differentiation of collagen fibers, slides were placed in a phosphomolybdic-phosphotungstic acid solution for 10–15 minutes or until collagen did not appear red in color. Without rinsing the slides, they were transferred to an aniline blue solution and stained for 5 minutes. Slides were finally rinsed with distilled water and rapidly dehydrated through 95% ethyl alcohol, absolute ethyl alcohol, and cleared in xylene then mounted. Slides were scanned using a Hamamatsu Nanozoomer Digital Pathology system (Hamamatsu City, Japan).

### Immunohistochemistry

Heart sections (following fixation and paraffin embedding) were stained for cardiac troponin-I (cTn-I) to evaluate any extent of damage to the structural integrity of the heart. To begin, slides were deparaffinized and placed in a pressure cooker and incubated for 20 minutes at 100°C(1 – addition of 500mL of deionized water to a pressure cooker; 2 – placement of slides in slide holder; covered with sodium citrate buffer). The slides were then allowed to cool and rinsed with deionized water. Endogenous peroxidase activities were inactivated in 3% H_2_O_2_ in TBS for 12 minutes at 4°C. Non-specific antibody binding sites were blocked, and slides were incubated with the primary antibody Troponin I (C-4): sc-133117 (Santa Cruz) overnight at 4°C; 1:500 dilution. After incubation, the slides were washed three times in 1X TBST for 3 minutes per wash. Secondary detection was performed by incubating the slides for 1 hour at room temperature using Goat-anti-mouse IgG2a antibody HRP. Next, the slides were washed, incubated with 3,3’-Diaminobenzidine for 5–10 minutes and washed once more in deionized water. Slides were now treated with hematoxylin, then dehydrated and mounted with a coverslip. Slides were scanned using a Hamamatsu Nanozoomer Digital Pathology system (Hamamatsu City, Japan). Prior to staining, positive and negative controls were established to ensure antibody-specific binding. Immunohistochemistry was supported by the measurement of cTn-I gene and protein.

### RNA Isolation and Quantitative Real-Time PCR

Approximately 50–100 mg of heart tissue was placed into 300 μL of TRIzol (MRC, Inc., Cincinnati, OH, USA) and homogenized using a hand-held homogenizer. After incubation for 5 minutes at room temperature, 30 μL of 1-bromo-3-chloropropane (Sigma-Aldrich, St. Louis, MO, USA) was added and vortexed. Samples were centrifuged at 12,000 rpm for 15 minutes at 4°C. The supernatant was transferred into a new tube for the addition of 70% ethanol (1:1). Total RNA was isolated using RNeasy mini kit (Qiagen, Germantown, MD, USA) according to the manufacturer’s protocol. Quantification of RNA was performed using a NanoDrop spectrophotometer (ThermoFisher Scientific, Waltham, MA, USA). Two micrograms of total RNA were reverse transcribed using oligo-(dT) 20 primers and M-MLV reverse transcriptase from Promega (Madison, WI) to synthesize complementary DNA. Gene expression was measured by real-time polymerase chain reaction (StepOne Real-Time PCR System; Applied Biosystems, Foster City, CA, USA) by measurement of SYBR Green. Messenger RNA (mRNA) concentrations were normalized to cyclophilin expression. The PCR primers and sequences are listed in Table 1.

### Measurement of Proteins

In addition to IHC, enzyme linked immunosorbent assay (ELISA) kits were used following the manufacturer’s protocol to measure cardiac *Tnfα*, *Il1β*, *cTn*-*I* (Abcam; Cambridge, MA), and transforming growth factor beta1 or *Tgfβ1* (Elabscience; Houston, TX).

### Statistical Analyses

Data is presented as the mean ± SE. One-way analysis of variance (ANOVA) was performed on data from the 1 st and 2 nd animal groupings, with diet and sitagliptin as the main effects, respectively. Two-way ANOVA was utilized for the third experiment using diet and sitagliptin as the main effects. Tukey’s test was used for multiple comparisons. Analysis was conducted using GraphPad Prism version 8.0.2 (San Diego, California).

## Results

### Effect of atherogenic diets on changes to cardiac biochemical parameters in male SD rats

Cho feeding resulted in significantly lowered *cTn*-*I* protein levels (~34%) compared to Con-fed rodents, while its gene expression was increased (50%); *1a*, *1b*. Biomarkers of inflammation (*Tnfα*, *Il11β*) and fibrosis (*Tgfβ1*), were affected in Cho-feeding as well, showing increases; *1a*, *1b*. The addition of Met to the Cho diet (MC) attenuated the adverse responses seen in Cho feeding, bringing them closer to baseline levels. The combination diet (MC), along with Met-feeding (alone), also increased serum *taurine*, a biomarker for antioxidation; 1c. Interestingly, the reduction of cTn-I protein was unanticipated and warranted further investigation for showing a diet-induced effect in the heart. The observed responses were independent of body composition, body weight and blood glucose levels, which were similar among dietary groups. Cardiac responses seen at this point appear to coincide with our hepatic studies, in that Cho feeding (alone) resulted in adverse effects [33, 34].

### Effect of sitagliptin on changes to cardiac biochemical parameters in male SD rats

With a second animal grouping, the cardioprotective potential of sitagliptin was investigated. Rats fed Con, Met, Cho, and MC diets were administered *sitagliptin* (*100 mg/kg/day*) by oral gavage; Con-S, Met-S, Cho-S, MC-S. An additional Con group was added to validate an expected null effect of drug with a normal diet - here, rats were gavaged with vehicle (water); Con-V. Ultimately, relative comparison was to Con-V for determining adverse effects, as there was little to no difference between Con-V and Con-S for any of the parameters measured.

In the 1st animal grouping, Cho feeding (alone) signficantly lowered *cTn*-*I* protein, increased its gene expression, and increased gene & protein expression of *Tnfα*, *Il1β*, and *Tgfβ1*. Met and MC feeding led to increases in serum taurine. With sitagliptin administration, the adverse cardiac responses seen in Cho feeding alone became exacerbated. Sitagliptin further increased *Tnfα*, *Il1β*, and *Tgfβ1* gene (~ 75–175%) and protein (~ 35–45%) in rats fed Cho; *2a*, *2b*. Cardiac troponin-I gene expression was further increased with drug administration as well by ~ *350%*, however, its protein levels were further reduced by approximately 10%. Sitagliptin increased *serum taurine* in Met and MC feeding, as was the case without the drug being administered; 2c. Alongside the effect of sitagliptin increasing serum taurine in Met and MC, gene & protein expression of *Tnfα*, *Il11β*, *Tgfβ1*, and *cTn*-*I* in both dietary groups were attenuated and brought closer to baseline levels. Consequently, the cardiac responses observed with sitagliptin administration were independent of body composition, body weight and blood glucose levels, which were similar among diet groups.

#### Effects of atherogenic diets and sitagliptin on cardiac structural and biochemical changes in male SD rats

Following independent observations of the effects of diet and sitagliptin in HChol, a 3rd experiment was conducted to further examine and corroborate those previous findings. Since Met feeding (alone) was shown to have a null effect on increasing adverse reponses in the heart with and without sitagliptin, it was omitted from this experiment.

Histopathological evaluation revealed both an increase in collagen deposition surrounding blood vessels in cardiac tissue (*3a*) and a reduction in myocardial striations (*4a*) in rats fed Cho. Administration of sitagliptin appears to exacerbate the effects of both to some degree. Similarly, gene and/or protein expression of related biomarkers followed similar patterns showing increases in pro-fibrotic responses (*αSMA*, *Tgfβ1*) and decreases in structural integrity (*cTn*-*I*); 3b, 4b. Pro-inflammatory markers (*Tnfα*, *Il1β*) and those related to Cho & fatty acid transport (*Lectin*-*like oxidized low*-*density lipoprotein* - *Lox*-*1*; *Rat fatty*-*acid*-*binding protein* - *rFABP*) were increased as well in HChol, and exacerbated with sitagliptin administration; 5a, 5b. Lectin-like oxidized low-density lipoprotein receptor is of importance because it is a scavenger receptor involved in oxidized low-density lipoprotein (oxLDL) uptake from the blood, after which oxLDL ultimately contributes to arterial plaque formation [39]. Rat fatty-acid-binding protein is part of a family of transport proteins that distribute fatty acids and other lipophilic compounds across intra- and extracellular membranes [40]. Addition of Met to the Cho diet (+/− sitagliptin) attenuated all adverse responses observed in HChol (+/− sitagliptin), bringing them closer to baseline levels. Serum taurine was the sole biomarker increased in MC, with and without sitagliptin administration; 5b. All responses were independent of body composition, body weight and blood glucose levels, which were similar among groups.

## Discussion

It is well known that CVD and NAFLD are major public health concerns globally with high morbidity and mortality. Both have been associated with elevated circulating levels of Cho and *Hcy*, an intermediate in Met metabolism. Though endogenous as well as dietary Cho sources contribute to circulating levels of Cho, non-pharmaceutical management (i.e., dietary approaches) is well-known for lowering Cho levels. With the emerging controversy about the role of Cho in CVD, it remains evident that elevated blood Cho can greatly affect liver function, as the liver is a main processing center for Cho [41]. Also, there are reports that point to the liver being affected by HChol more than the heart and that chronic liver disease can have a direct impact on heart function [42].

Our experimental approach was to feed a dietary excess of Cho and Met independently, but moreso in combination since studies on the combined effects are not as numerous. We also aimed to investigate the cardioprotective potential of sitagliptin, which is documented as improving cardiac function and ejection fraction. Sitagliptin is used in pharmacotherapy of glucose management in type II diabetics and has displayed positive effects (e.g., weight lowering, reduction of inflammation / oxidative stress / fibrotic responses) independent of glucose-lowering [43,44, 45].

Adverse biochemical events occurring in the heart can result in conditions like arrhythmias, myocardial infarction, and heart failure eventually [46, 47, 48]. Diagnosis of such events requires a concerted effort that usually commences with cardiac function tests. This involves imaging techniques (e.g., echocardiograms, magnetic resonance imaging scans, computed tomography scans, nuclear cardiac stress test, coronary angiogram or left heart catheterization, X-rays, etc.), biopsies, and/or serological assays [49, 50, 51, 52, 53]. As it relates to a clinical diagnosis of acute myocardial infraction, elevated blood cTn-I protein levels are what typically aids that determination. It serves as indicator of cardiac structural damage [53, 54]. In our animal study, we assessed cardiac function primarily by histopathological evaluation and quantification of cTn-I protein in heart tissue. *Cho feeding resulted in a significant loss of the protein*, *an effect that was exacerbated with sitagliptin* - *a novel finding contrary to our central hypothesis*, *which was to anticipate such an effect in MC*-*feeding and without sitagliptin administration*. Similarly, Han et al. (2018) saw a reduction of cTn-I protein in heart tissue as a result of feeding male SD rats a high-fat, high Cho diet for 14 and 28 days. Interestingly, we observed an approximate 350% increase to cTn-I gene expression in HChol (+ sitagliptin), an effect that is possibly due to a compensatory response, as demonstrated by Sasse et al. (1993). Studies by Packer (2018) also show a positive correlation between DPP-4 inhibitor use and adverse cardiac events, citing their ability to cause and/or worsen heart failure. Rouse et al. (2014) and Shahbaz et al. (2018) correlate sitagliptin use with pancreatic injury and acute hepatitis, respectively.

Cho feeding in our study was shown to increase collagen deposition surrounding blood vessels of the heart, as well as within the interstitial spaces. Sitagliptin appears to exacerbate the effect to some degree. Notably, cardiac fibrosis is classified as either endomyocardial fibrosis, infiltrative & reactive interstitial fibrosis, or replacement fibrosis [61]. HChol (+/− sitagliptin) seems to have resulted in a form cardiac perivascular fibrosis, which is characterized by collagen accumulation around blood vessels [62, 63]. This is known to precede or coincide with *reactive interstitial fibrosis* - collagen accumulation that causes expansion of cardiac interstitial spaces with minor cardiomyocyte loss [62, 63]. Although the increase in collagen deposition by Cho may not seem unique, as this was demonstrated by Han et al. (2018), the seemingly sitagliptin exacerbation is interesting. A reason for such an observation could be due to sitagliptin’s interaction with Cho that affects some factor in Tgfβ signaling. Three isotypes of Tgβ have been identified in mammals (Tgβ1, Tgββ2, Tgfβ3) and many animals studies identify *type* 1 as the “master regulator” that promotes fibrotic development in several tissues [64, 65, 66]. Tgfβ1 utilizes several signaling pathways to elicit a variety of actions (e.g., autophagy, differentiation, apoptosis, and cellular proliferation). However, it is the Smad-dependent (canonical) pathway that is most noted as resulting in fibrosis [64, 65, 66]. Sitagliptin could possibly stimulate the canonical pathway in some way, but this remains to be proven. Similar to cardiac smooth muscle, myofibroblasts in heart tissue express αSMA and are abundantly located in the thick myocardial layer. Myofibroblasts help to regulate various functions such as matrix deposition & degradation and growth-factor secretion [67]. Expression of αSMA and Tgβ1 (gene and/or protein) was increased in HChol as well and exacerbated with sitagliptin.

Insight into the underlying molecular mechanisms by which adverse structural responses were seen in HChol, with and with sitagliptin administration, was investigated in our study. Biochemical changes are those precede structural changes in all cell types and are related to processes like oxidative stress and inflammation [68, 69]. Such changes could, in fact, be sex-specific, as Marques et al. (2018) discovered an association between increased IL-6 and C-reactive protein expression and the development of interstitial myocardial fibrosis in men. Additional literature also points to Tnfα and IL-1/6 being key mediators for myocardial alterations [71]. Serum Cho was increased approximately 100% in our rats due to Cho feeding - sitagliptin had no added effect on either decreasing or increasing serum Cho. This, however, resulted in a substantial increase in hepatic gene and protein expression of several biomarkers related to inflammation and oxidative stress, when rats were administered sitagliptin; *Pathak et al*. (*2019*), *Kumar et al*. (*2020*). Even though we observed significant increases to pro-inflammatory, pro-fibrotic, and Cho/fatty acid transport biomarkers in the heart (+ sitagliptin), they are far outweighed by those in the liver. This could be due to the liver’s increased exposure to compounds in the blood, since it primarily functions to metabolize, transport, and filter compounds that are absorbed and placed into circulation [72]. In either case of the liver or heart, sitagliptin was shown to enhance the adverse biochemical responses seen in HChol.

The addition of Met to the Cho diet did not produce an added adverse effect as originally hypothesized by our group. In fact, it proved beneficial by way of attenuating all adverse cardiac responses in HChol, bringing them closer to normal levels. This was interesting because Met restriction is outlined in literature as being beneficial, however, our results were on the contrary. We did not notice any obvious disruptions to Met metabolism, as Hcy levels and gene expression of the Met-metabolizing enzymes were unaffected. Unexpectedly, Met and MC feeding led to increased serum taurine levels. Taurine is a compound with anti-oxidative and anti-inflammatory effects, both of which could have contributed to the beneficial responses we observed [73]. In addition to taurine, there are other intermediates in Met metabolism that are documented to elicit multiple health benefits, i.e., anti-oxidation & -inflammation, vasodilation [73, 75]. Additional studies are needed to better understand this.

In summary, our study provides insight into the effects of DPP4-inhibitor use and atherogenic diets on the biochemical and structural changes of the heart. We demonstrated that sitagliptin administration exacerbates adverse cardiac responses seen in HChol, while also revealing the beneficial potential of high dietary Met to attenuate such effects. For a better understanding of this diet-drug relationship, additional studies are needed. The beneficial aspect of high dietary Met observed in our study merit mechanistic understanding for exploring future therapeutic options considering the public health relevance of CVD and are thus translational.

## Figures and Tables

**Figure 1:**
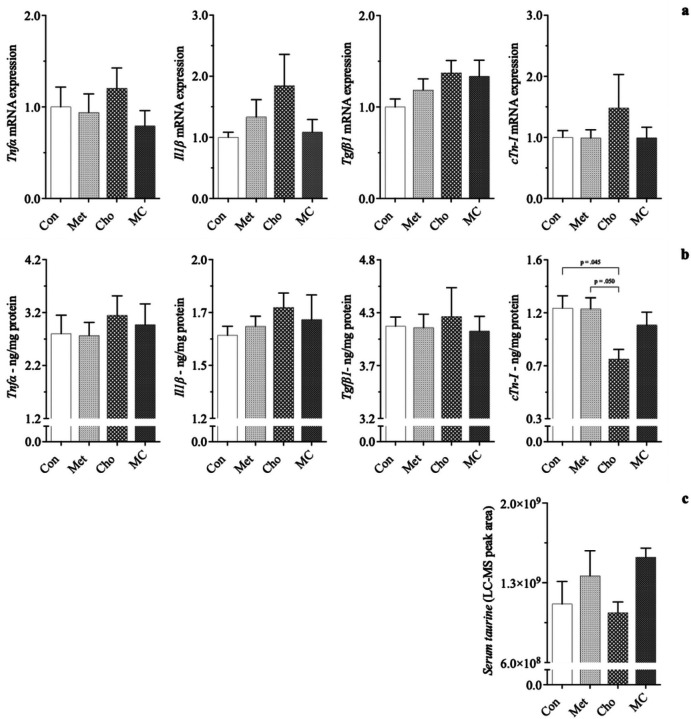
Effect of atherogenic diets on expression of cardiac biomarkers in male Sprague-Dawley rats. Rodents were fed either a Con, high Met, high Cho, or high Met + high Cho (MC) diet *ad libitum* for 35 days. Relative *gene* (*a*) *and protein* (*b*)*expression of pro*-*inflammatory* (*Tnfα*, *IIT*) *and pro*-*fibrotic* (*Tgfβ1*)*indicators are shown*, *along with serum taurine* (*c*), a biomarker that acts as an antioxidant. Values are presented as mean ± SE; n=7.

**Figure 2:**
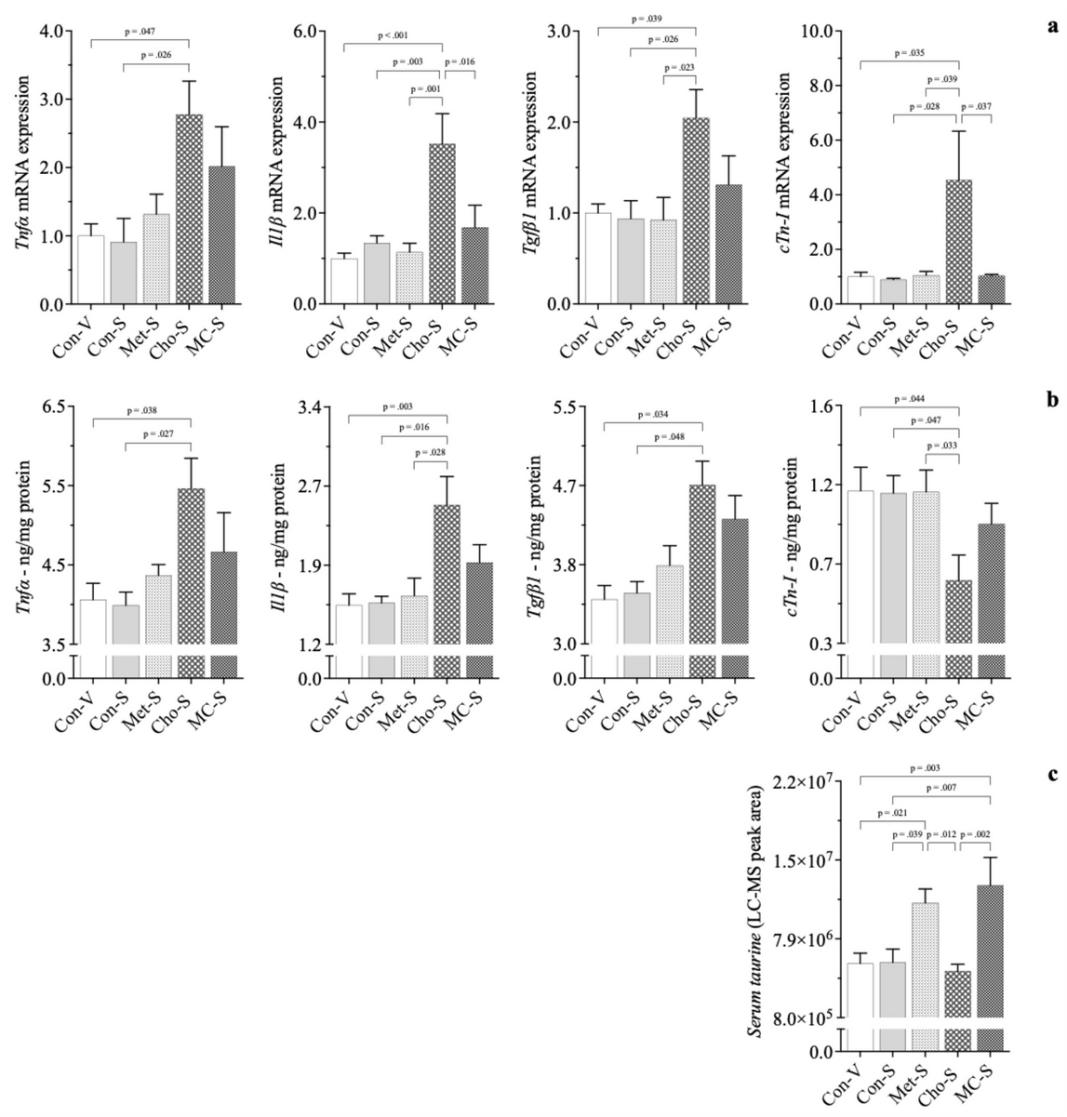
Effect of sitagliptin on expression of cardiac biomarkers in male Sprague-Dawley rats fed atherogenic diets. Rodents were fed either a Con, high Met, high Cho, or high Met + high Cho (MC) diet *ad libitum* for 35 days. Day 10 through 35, half the Con and all rats in Met, Cho, and MC groups were administered an aqueous suspension of *sitagliptin* (*100 mg/kg/day*) by oral gavage; Con-S, Met-S, Cho-S, MC-S. The remaining Con rodents were gavaged with vehicle; Con-V. Relative gene (*a*) *and protein* (*b*) expression of pro-inflammatory (*Tnfα*, *Il1β*) and pro-fibrotic (*Tgfβ1*) indicators are shown, along with *serum taurine* (*c*). Values are presented as mean ± SE; n=7.

**Figure 3:**
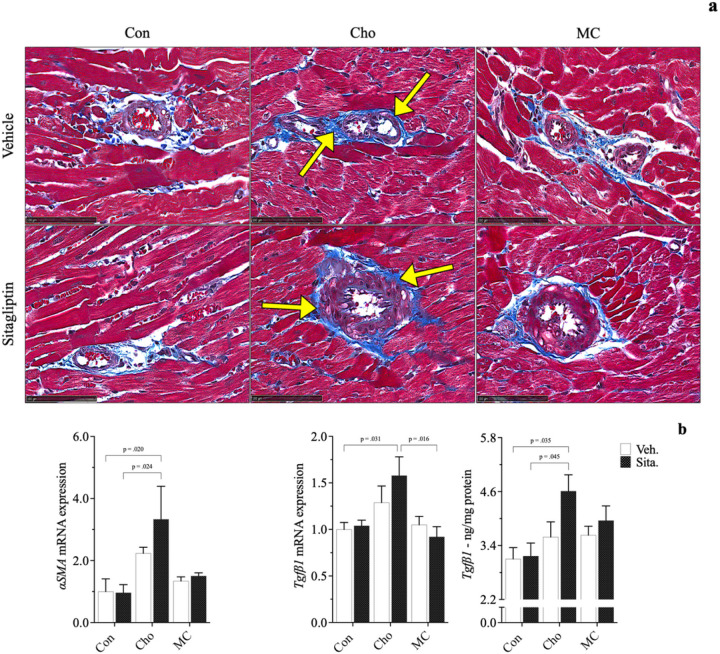
Effects of diet and sitagliptin on structural and biochemical parameters of fibrosis. Six-week-old male Sprague-Dawley rats were fed either a Con, high Cho, or MC diet *ad libitum*for 35 days. Day 10 through 35, half the rats in each group were administered an aqueous suspension of *sitagliptin* (*100mg/kg/day*) by oral gavage, while the remaining half were gavaged with vehicle (water). Representative photomicrographs of Masson’s trichrome staining (*a*; *40X*) of heart tissue showing collagen deposition (*arrows*), along with associated biomarkers of fibrosis (*b*), are depicted. Images show increased collagen deposition as a result of Cho feeding with and without sitagliptin administration. Values are presented as mean ± SE; n=8.

**Figure 4:**
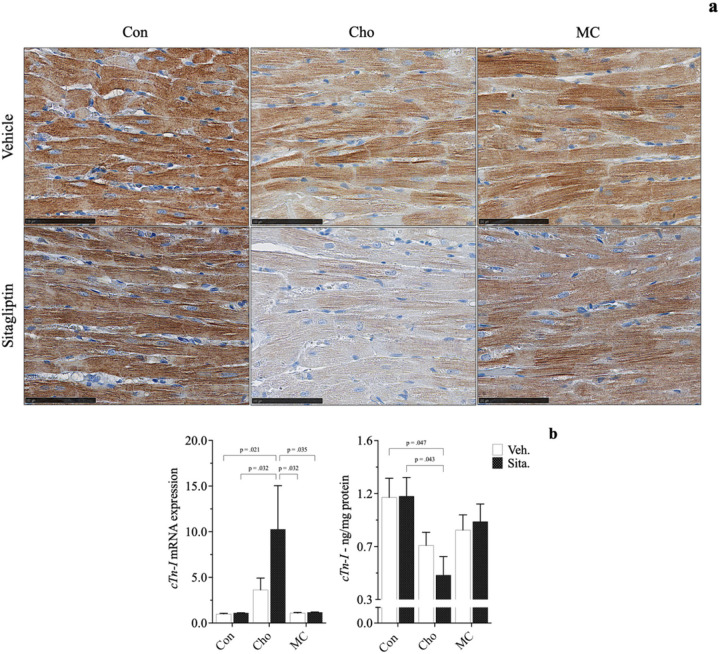
Effects of diet and sitagliptin on cardiac troponin-I expression in male SD rats. Six-week-old male Sprague-Dawley rats were fed either a Con, high Cho, or MC diet *ad libitum*for 35 days. Day 10 through 35, half the rats in each group were administered an aqueous suspension of *sitagliptin* (*100mg/kg/day*) by oral gavage, while the remaining half were gavaged with vehicle (water). Representative photomicrographs of H&E counterstain for *cTn*-*I* (*a*; *40X*) showing a reduction in myocardial striations (i.e., loss of protein) is shown, along with *cTn*-*I* gene expression and protein levels (*b*). Values are presented as mean ± SE; n=8.

**Figure 5:**
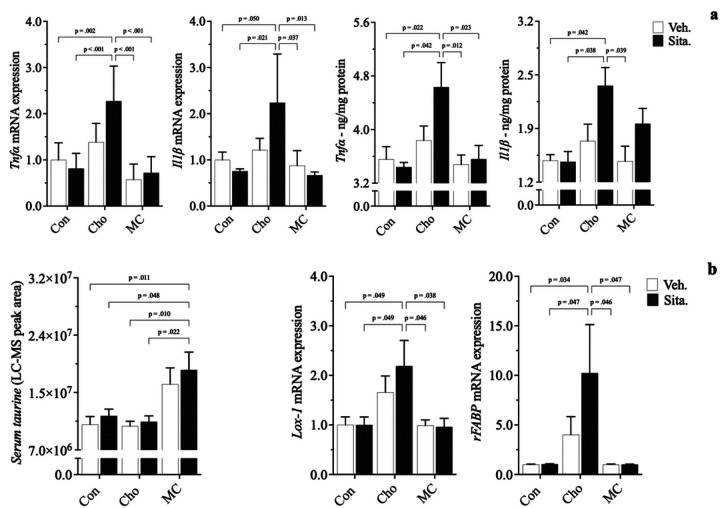
Effects of diet and sitagliptin on cardiac biomarkers of inflammation, Cho transport, and antioxidation. Six-week-old male Sprague-Dawley rats were fed either a Con, high Cho, or MC diet *ad libitum* for 35 days. Day 10 through 35, half the rats in each group were administered an aqueous suspension of *sitagliptin* (*100mg/kg/day*)by oral gavage, while the remaining half were gavaged with vehicle. Relative gene expression and protein levels of pro-inflammatory indicators in HChol (+/− sitagliptin) are shown (*a*), along with biomarkers of Cho/fatty acid transport and antioxidation (*b*). Values are presented as mean ± SE; n=8.

**Table 1:** Sequence of primers used for RT-qPCR.

Target Gene	Sequence
*CypA* Forward Reverse	TATCTGCACTGCCAAGACTGAGTGCTTCTTGCTGGTCTTGCCATTCC
*cTn-I* Forward Reverse	CACCTCAAGCAGGTGAAGAATCTTTCGGCCTTCCATTCC
*rFABP* Forward Reverse	CGGTACCTGGAAGCTAGTGGTCATCTGCTGTGACCTCGTC
*Il1β* Forward Reverse	CAAGCAACGACAAAATCCCTGGACAAACCGCTTTTCCATCTTC
*Lox1* Forward Reverse	CCCACAAGTCACAGACTCTTCCACACACTCACACACACAAATAC
*αSMA* Forward Reverse	GCTCCTCCAGAACGCAAATACAGCTTCGTCATACTCCTGTTT
*Tgfβ1* Forward Reverse	AGAGCCCTGGATACCAACTACAACCCAGGTCCTTCCTAAAG
*Tnfα* Forward Reverse	AGACCCTCACACTCAGATCAGTCTTTGAGATCCATGCCATTG

## Data Availability

The datasets used and/or analysed during the current study are available from the corresponding and first authors on reasonable request.

## Abbreviations

ANOVA: Analysis of variance
Cho: Cholesterol
CO_2_: Carbon dioxide
Con: Control
cTn-I: Cardiac troponin-I
CVD: Cardiovascular disease
DPP-4: Dipeptidyl peptidase-4
ELISA: Enzyme linked immunosorbent assay
rFABP: rat Fatty-acid-binding Protein
H_2_O_2_: Hydrogen peroxide
HChol: Hypercholesterolemia
Hcy: Homocysteine
HRP: Horseradish peroxidase
lgG2a: Immunoglobulin 2 alpha
II1β: Interlukin-1 beta
IL-6: Interlukin-6
LDL: Low-density lipoprotein
Lox-1: Lectin-like oxidized low-density lipoprotein receptor-1
MC: Methionine + Cholesterol
Met: Methionine
mg: Milligram
mL: Milliliter
mRNA: Messenger ribonucleic acid
NAFLD: Non-alcoholic fatty liver disease
NMR: Nuclear magnetic resonance
oxLDL: oxidized low-density lipoprotein
PCR: Polymerase chain reaction
rpm: Revolutions per minute
SEM: Standard error of the mean
αSMA: alpha-Smooth muscle actin
SYBR: Synergy Brands
TBS: Tris buffered saline
TBST: Tris buffered saline – Tween 20
Tgfβ1: Transforming growth factor beta 1
Tnfα: Tumor necrosis factor alpha
RNA: Ribonucleic acid
μL: Microliter
μm: Micrometer
